# Non-Viral Gene Therapy in Trabecular Meshwork Cells to Prevent Fibrosis in Minimally Invasive Glaucoma Surgery

**DOI:** 10.3390/pharmaceutics14112472

**Published:** 2022-11-16

**Authors:** Jinyuan Luo, Greymi Tan, Kai Xin Thong, Konstantinos N. Kafetzis, Neeru Vallabh, Carl M. Sheridan, Yusuke Sato, Hideyoshi Harashima, Aristides D. Tagalakis, Cynthia Yu-Wai-Man

**Affiliations:** 1 Faculty of Life Sciences & Medicine, King’s College London, London SE1 7EH, UK; 2 Department of Ophthalmology, Renmin Hospital of Wuhan University, Wuhan 430060, China; 3 Department of Biology, Edge Hill University, Ormskirk L39 4QP, UK; 4 Department of Eye and Vision Science, Institute of Life Course and Medical Sciences, University of Liverpool, Liverpool L69 3BX, UK; 5 Faculty of Pharmaceutical Sciences, Hokkaido University, Kita-12, Nishi-6, Kita-ku, Sapporo 060-0812, Japan

**Keywords:** nanoparticle, gene therapy, trabecular meshwork, fibrosis, MIGS

## Abstract

The primary cause of failure for minimally invasive glaucoma surgery (MIGS) is fibrosis in the trabecular meshwork (TM) that regulates the outflow of aqueous humour, and no anti-fibrotic drug is available for intraocular use in MIGS. The myocardin-related transcription factor/serum response factor (MRTF/SRF) pathway is a promising anti-fibrotic target. This study aims to utilise a novel lipid nanoparticle (LNP) to deliver MRTF-B siRNA into human TM cells and to compare its effects with those observed in human conjunctival fibroblasts (FF). Two LNP formulations were prepared with and without the targeting peptide cΥ, and with an siRNA concentration of 50 nM. We examined the biophysical properties and encapsulation efficiencies of the LNPs, and evaluated the effects of *MRTF-B* silencing on cell viability, key fibrotic genes expression and cell contractility. Both LNP formulations efficiently silenced *MRTF-B* gene and were non-cytotoxic in TM and FF cells. The presence of cΥ made the LNPs smaller and more cationic, but had no significant effect on encapsulation efficiency. Both TM and FF cells also showed significantly reduced contractibility after transfection with MRTF-B siRNA LNPs. In TM cells, LNPs with cΥ achieved a greater decrease in contractility compared to LNPs without cΥ. In conclusion, we demonstrate that the novel CL4H6-LNPs are able to safely and effectively deliver MRTF-B siRNA into human TM cells. LNPs can serve as a promising non-viral gene therapy to prevent fibrosis in MIGS.

## 1. Introduction

Glaucoma is the leading cause of irreversible blindness worldwide, affecting 79.6 million individuals in 2020, and the prevalence is estimated to rise to 111.8 million by 2040 [1,2]. Although the underlying mechanism of glaucoma remains unclear, high intraocular pressure (IOP) is an important risk factor [3]. Surgery to lower IOP is the main treatment modality in medically uncontrolled glaucoma, but still faces the challenges of frequent drug administration and surgical failure due to scarring [4].

**Figure 1:**
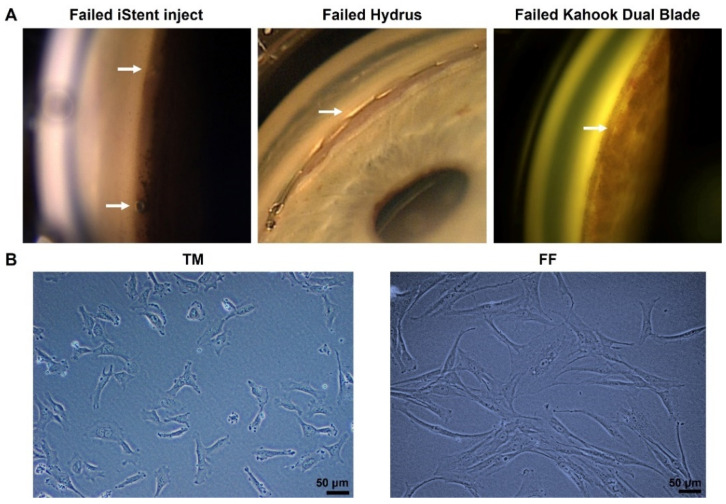
(**A**) Failed minimally invasive glaucoma surgery (MIGS) due to fibrosis. The arrow indicates scarring in iStent inject (**left**), Hydrus (**middle**), and the fibrotic membrane over the trabecular meshwork (TM) after Kahook Dual Blade surgery (**right**). (**B**) Phase-contrast microscopy images of human trabecular meshwork cells (TM, **left**) and human conjunctival fibroblasts (FF, **right**) cultured on plastic dishes. Scale bar, 50 µm.

Minimally invasive glaucoma surgery (MIGS), such as iStent, Hydrus, and Kahook Dual Blade, represent a group of glaucoma drainage devices and procedures that have recently been developed to provide a safer technique of reducing IOP [5]. Despite a micro-invasive approach and minimally induced tissue trauma, the main cause of failure in MIGS is fibrosis around implants in the trabecular meshwork (TM) [6,7] (Figure 1A). Mitomycin-C (MMC) and 5-fluorouracil (5-FU) are too toxic to be used inside the eye and no anti-fibrotic drugs are available to be used intraocularly to prevent fibrosis in TM tissues. There is thus a large unmet clinical need to develop a novel, targeted, and non-cytotoxic anti-fibrotic therapy in MIGS.

Serum response factor (SRF) and its co-activators, myocardin-related transcription factors A and B (MRTF-A and MRTF-B), are major regulators of cytoskeletal gene expression [8]. Glaucoma surgery creates tissue injury and upregulates the level of transforming growth factor beta (TGF-β) at inflammatory sites, further stimulating myofibroblast transformation and extracellular matrix (ECM) remodeling, eventually resulting in conjunctival fibrosis [9]. It has been shown that the presence of MRTFs is essential for TGF-β-induced fibrosis [10,11], making the MRTF/SRF pathway a promising therapeutic target for new anti-fibrotic therapeutics.

TM tissues are critical for regulating the outflow of aqueous humour (AH). In glaucoma patients, the ECM structures of TM tissues are deformative [12,13], and there is a positive correlation between stiff TM tissues and elevated IOP [14]. These TM cells have contractile properties similar to conjunctival fibroblasts. The Rho GTPase/Rho kinase signaling is reported to play an important role in TGF-β2 induced fibrogenic activity and the expression of various biomarkers of myofibroblasts in human TM cells. Importantly, these changes were found to be mediated by the MRTF/SRF pathway [15,16]. The activation of RhoA, TGF-β2 and MRTF-A induces dysregulated accumulation of ECM, which is closely linked to TM cell contractility and AH outflow [15,17]. However, whether the MRTF-B/SRF pathway specifically plays a role in TM tissue contraction has not been proven and thus warrants further investigation.

Small interfering RNAs (siRNAs) are double-stranded RNA molecules (20–25 nucleotides) that regulate gene expression by binding to their target mRNAs through complementary sequences, resulting in their degradation [18,19]. Ideal carrier systems are required to deliver the therapeutic molecules, such as siRNA, mRNA or plasmid DNA, as nucleic acids are susceptible to breakdown in biological fluids and cannot efficiently access or accumulate at intracellular sites even if they reach their target cells [20,21]. Lipid nanoparticles (LNPs) designed with cationic lipids are efficient vehicles for siRNA delivery. Unlike viral vectors and other non-viral delivery systems, LNPs are relatively simple to synthesise, easy to modify, and economical to manufacture [22,23]. The interplay between charge repulsion and steric stabilisation of the lipid membrane interface leads to the nanochannel formation in the internal structure of LNPs, and nucleic acids are encapsulated inside the LNPs and protected from enzymatic degradation and rapid elimination [24,25]. The head-group of cationic lipids with an acid dissociation constant value of 7 assures low toxicity of LNPs both *in vitro* and *in vivo* [26]. Additionally, the multiple amines in the head-group also improve the adherence and release of siRNAs when penetrating through the biological membrane of cells [27].

In our previous studies [28,29], we have demonstrated that both non-PEGylated and PEGylated nanoparticles can efficiently deliver MRTF-B siRNAs into human conjunctival fibroblasts (FF) with no cytotoxicity. A cleavable targeting peptide cΥ was utilised to achieve targeted high-specificity delivery of siRNAs. The peptide cΥ is composed of a positively charged nucleic acid binding domain of 16 lysines (K_16_), a GA spacer, a targeting domain (CYGLPHK), and a cleavable RVRR linker recognised by endosomal enzymes. Results showed that the addition of the targeting peptide cΥ improved the encapsulation efficiency of the LNP formulations in FF cells [28].

In this study, we further used this novel LNP, containing the cationic lipid CL4H6 and the targeting peptide cΥ, in order to deliver MRTF-B siRNA *in vitro* into human TM cells to efficiently silence *MRTF-B* gene expression, and to compare its effects with those observed in FF cells. We demonstrated, for the first time, the role of *MRTF-B* silencing in TM cell contractility, and investigated the effects of the addition of the targeting peptide cΥ on the physicochemical properties, efficacy, and safety profile of the LNP formulations.

## 2. Materials and Methods

### 2.1. Materials

CL4H6, a pH-sensitive cationic lipid, was synthesised as previously described [30]. 1,2-Dioleoyl-sn-glycero-3-phosphoethanolamine (DOPE) and 1,2-dimirystoyl-rac-glycero, methoxyethylene glycol 2000 ether (PEG-DMG) were obtained from Sigma-Aldrich (St. Louis, MO, USA). Cleavable peptide cΥ was acquired from AMS Biotechnology (Abingdon, UK). MRTF-B siRNA and irrelevant control siRNA were purchased from Horizon Discovery (Cambridge, UK), and their sequences are shown in Table 1.

### 2.2. Cell Culture

Human TM cells were cultured from trabecular meshwork tissues and human FF cells were cultured from conjunctival tissues of glaucoma patients after informed consent. All experiments were carried out following the rules of the Declaration of Helsinki and approved by the West of Scotland Research Ethics Committee (REC 19/WS/0146). SV40-immortalized (NTM5) human TM cells were obtained from Alcon (Fort Worth, TX, USA), which have been characterised and used in previous studies [31]. The conjunctival fibroblasts were cultured from a piece of conjunctival tissue from a 63-year-old female patient with glaucoma. The conjunctival tissue was mechanically dispersed and the tissue fragments were placed in Dulbecco’s modified Eagle’s medium (DMEM) (Gibco, Thermo Scientific, UK), 10% fetal calf serum, 100 units/mL penicillin, and 0.1 mg/mL streptomycin. After the outgrowth from the explant, the FF cells were trypsinised and cultured in the above medium. The cells were grown in an incubator at 37 °C with 5% CO_2_ and 95% humidity.

### 2.3. Nanoparticle Formulations

A 90% t-BuOH solution containing CL4H6, DOPE and PEG-DMG was prepared at a molar ratio of 50:50:1 (1.4 mg/mL total lipid concentration), followed by the addition of 40 μg siRNA to give an N/P ratio of 7.5. The LNPs were prepared in 20 mM MES buffer with pH 6.0 and stored at 4 °C as previously described [28,30]. The LNP + cΥ nanoparticles were synthesised by mixing LNPs and cΥ at a weight ratio of 4:1.

### 2.4. Nanoparticle Size and Zeta Potential

Dynamic light scattering (DLS) and laser Doppler anemometry were used to measure the size and zeta potential, respectively, of the LNPs using a Nano ZS Zetasizer (Malvern Instruments, Malvern, UK). The specifications were set as previously described [29]: automatic sampling time, 10 measurements per sample; refractive index, 1.330; dielectric constant, 78.5; viscosity, 0.8872 cP; temperature, 25 °C. Zeta potential settings were calibrated against the standard (−68 ± 6.8 mV). Measurements for each sample were repeated in triplicates and were analysed using DTS version 5.03.

### 2.5. Transmission Electron Microscopy (TEM)

A 300-mesh copper grid coated with Formvar/carbon support film (Agar Scientific, Stanstead, UK) was used to load the LNPs. After incubation for 3 min at room temperature, the samples were negatively stained with 1% uranyl acetate for 15 s and then blotted with filter paper and air dried. The samples were viewed using a Philips CM120 BioTwin TEM (FEI Company, Hillsboro, OR, USA) with an accelerating voltage of 120 kV, and the images were captured by an AMT 5MP digital TEM camera (Deben UK, Suffolk, UK).

### 2.6. Encapsulation Assay

The final concentration and encapsulation efficiency of each LNP were determined using the RiboGreen assay kit (ThermoFisher Scientific, Loughborough, UK). The assay was carried out by diluting 100 μL of each LNP in 100 μL of 10 mM HEPES buffer at pH 7.4 containing 20 μg/mL dextran sulfate and RiboGreen solution, with or without the presence of 0.1 *w*/*v*% Triton X-100 (Sigma Aldrich, Gillingham, UK). The standard curve was established by diluting the siRNA-only solutions under the same procedure and used for calculating the concentrations of the LNPs. Fluorescence was measured using a FLUOstar Omega (BMG LABTECH, Aylesbury, UK) at λex = 500 nm and λem = 525 nm [28,32]. The siRNA encapsulation efficiency was calculated by comparing the siRNA concentrations with and without the presence of Triton X-100, using the formula:

$$\mathrm{siRNA}\;\mathrm{encapsulation}\;\%=\frac{\mathrm{Encapsulated}\;\mathrm{siRNA}-\mathrm{Unencapsulated}\;\mathrm{siRNA}}{\mathrm{Total}\;\mathrm{siRNA}\;\mathrm{concentration}}\times 100$$

### 2.7. In Vitro Transfection

Human TM and FF cells were seeded into 6-well plates at a density of 1 × 10^5^ cells per well. After 24 h, TM and FF cells were incubated with four different LNPs for 48 h in complete culture media before further experiments: LNP-MRTF-B siRNA, LNP-MRTF-B siRNA + cΥ, LNP-Control siRNA, LNP-Control siRNA + cΥ.

### 2.8. Real-Time Quantitative PCR

The RNeasy mini kit (Qiagen, Crawley, UK) and high-capacity cDNA reverse transcription kit (ThermoFisher Scientific, Loughborough, UK) were used for total RNA extraction and cDNA synthesis, respectively, according to the manufacturer’s protocol. A QuantiFast SYBR Green PCR kit (Qiagen, Crawley, UK) was performed on a ViiA7 Real-Time PCR system (ThermoFisher Scientific, Loughborough, UK) for RT-qPCR assay. The reaction conditions were as follows: stage 1, 95 °C for 5 min; stage 2, 95 °C for 10 s; stage 3, 60 °C for 30 s; repeated 40 times. The primers used were as follows: *MRTF-B*, 5′-CTTCCTGTGGACTCCAGTG-3′, 3′-TGTGACTCCTGACTCGCAG-5′; *ACTA2*, 5′-AATGCAGAAGGAGATCACGC-3′, 3′-TCCTGTTTGCTGATCCACATC-5′; *COL1A2*, 5′-TGGATGAGGAGACTGGCAAC-3′, 3′-TTAGAACCCCCTCCATCCCAC-5′; *GAPDH*, 5′-ACGGATTTGGTCGTATTGGGC-3′, 3′-TTGACGGTGCCATGGAATTTG-5′. Each experiment was performed in triplicates. The following formula was used to calculate relative gene expression:

$$\mathrm{Relative}\;\mathrm{gene}\;\mathrm{expression}=2^{-\left({\Delta \mathrm{Ct}}_{\mathrm{target}}-{\Delta \mathrm{Ct}}_{\mathrm{GAPDH}}\right)}$$

### 2.9. Cell Viability Assay

Human TM and FF cells were seeded into 96-well plates at a density of 6.25 × 10^3^ cells per well. Each LNP was added and all conditions were repeated in triplicates. After 24 h, the media in each well were replaced by 100 μL of fresh culture media, and 20 μL of the Cell Titer 96 Aqueous one solution (Promega, Southampton, UK) were added to each well, according to the manufacturer’s instructions. The plate was placed for 2 h in the incubator, and the absorbance was measured at 540 nm using a FLUOstar Omega (BMG LABTECH, Aylesbury, UK). The cell viability of untreated control cells was normalised to 100%, and the cell viability of other groups was calculated as a percentage compared to untreated control cells.

### 2.10. Collagen Contraction Assay

Human TM and FF cells were trypsinised and counted. A cell suspension of 1 × 10^5^ cells was centrifuged for 5 min at 1500 rpm. The supernatant was removed and 100 μL of fetal calf serum were added to resuspend the cell pellet. A collagen solution was prepared by 1 mL of Type 1 collagen and 160 μL of concentrated media, and adjusted to pH 7.0 as previously described [28]. The cells were mixed with the collagen solution and incubated for 10 min at 37 °C with 5% CO_2_ and 95% humidity in MatTek dishes (MatTek Life Science, Ashland, MA, USA). After carefully releasing the gel, 2 mL of growth media was added to each MatTek dish. Images were recorded once the polymerised matrices were released (*t*_0_) and daily over the following 7 days (*t*_n_). Data were analysed using the Image J software version 1.53t and the following formula was used to calculate the gel surface area:

$$A\left(t_{n}\right)\;in\;\%=100-\left(100\times \frac{r_{t_{n}}{}^{2}}{r_{t_{0}}{}^{2}}\;\right)$$

where *A* is the gel surface area (this value was normalised to the value calculated at *t*_0_) and *r* is the radius. Each experiment was repeated in triplicates.

### 2.11. Statistical Analysis

All data are expressed as mean ± SEM. After analysing the normality of the data, statistical analysis was carried out using One-way ANOVA followed by Tukey’s or LSD post hoc test. A *p* value < 0.05 was considered as statistically significant.

## 3. Results

### 3.1. Cell Morphology of Human Trabecular Meshwork (TM) and Conjunctival Fibroblast (FF) Cells Assessed by Phase Contrast Microscopy

Both TM and FF cells were cultured in the same culture media and passaged using the same aseptic technique. The TM cells could reach confluency (~90%) after only 2 days in culture. On the other hand, the FF cells grew much slower, reaching confluency after a week in culture with media changed every three days. Noticeably, they also had different morphology. The FF cells had a longer spindle-like cell shape compared to the TM cells, which had a more rounded shape and a shorter length (Figure 1B).

### 3.2. Biophysical Characteristics and Encapsulation Efficiencies of Lipid Nanoparticles (LNPs)

**Figure 2:**
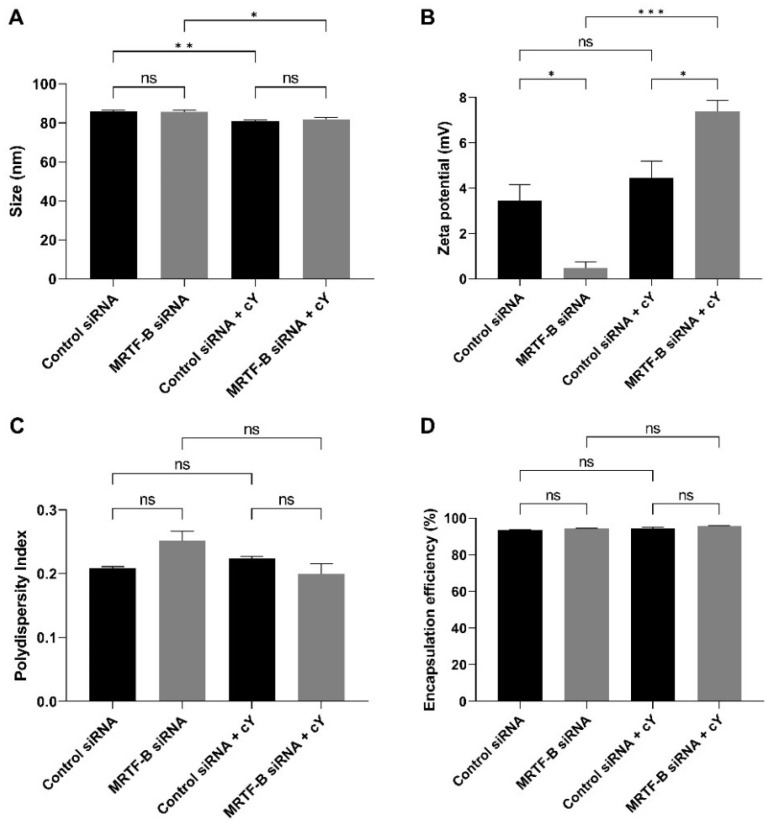
Biophysical properties and encapsulation efficiencies of different lipid nanoparticles (LNPs). (**A**) Size in nm. (**B**) Zeta potential in mV. (**C**) Polydispersity index. (**D**) % Encapsulation efficiency. Results represent mean ± SEM. *n* = 3. *, *p* < 0.05; **, *p* < 0.01; ***, *p* < 0.001; ns, not significant.

The average sizes of LNP-Control siRNA and LNP-MRTF-B siRNA were 85.80 ± 0.76 nm and 85.68 ± 0.89 nm, respectively, with no statistically significant difference (*p* > 0.99). The LNP-Control siRNA + cΥ and LNP-MRTF-B siRNA + cΥ also had similar average sizes of 80.98 ± 0.51 nm and 81.90 ± 0.79 nm, respectively (*p* = 0.82). With the addition of cΥ, the LNP-Control siRNA + cΥ and LNP-MRTF-B siRNA + cΥ had significantly smaller average sizes when compared with the LNP-Control siRNA (*p* = 0.008) and LNP-MRTF-B siRNA (*p* = 0.03) without cΥ (Figure 2A).

All formulations were near neutral or weakly cationic. The zeta potentials of LNP-Control siRNA and LNP-MRTF-B siRNA were 3.45 ± 0.71 mV and 0.48 ± 0.28 mV, respectively (*p* = 0.03). The LNP-Control siRNA + cΥ and LNP-MRTF-B siRNA + cΥ were weakly cationic with zeta potential values of 4.46 ± 0.74 mV and 7.39 ± 0.49 mV, respectively (*p* = 0.03). The presence of cΥ made both LNP-MRTF-B siRNA + cΥ (*p* < 0.001 versus its counterpart with no peptide) and LNP-Control siRNA + cΥ (*p* = 0.63 versus its counterpart with no peptide) more cationic (Figure 2B).

All polydispersity indexes (PDI) were less than 0.3, indicating a homogenous particle size distribution. The PDI of LNP-Control siRNA, LNP-MRTF-B siRNA, LNP-Control siRNA + cΥ and LNP-MRTF-B siRNA + cΥ were 0.209 ± 0.002, 0.251 ± 0.016, 0.223 ± 0.004 and 0.199 ± 0.017, respectively. There were no statistically significant differences between them or between LNPs with and without cΥ (Figure 2C).

Furthermore, the presence of cΥ increased the drug encapsulation efficiency by a small margin but with no statistical significance. All four LNPs had similar high encapsulation efficiencies. The encapsulation efficiency of LNP-Control siRNA was 93.5 ± 0.2%, whereas that of LNP-MRTF-B siRNA was 94.3 ± 0.2% with no statistically significant difference. Similarly, the encapsulation efficiencies of LNP-Control siRNA + cΥ and LNP-MRTF-B siRNA + cΥ were 94.2 ± 0.9% and 95.8 ± 0.2%, respectively, with no statistically significant difference (Figure 2D).

**Figure 3:**
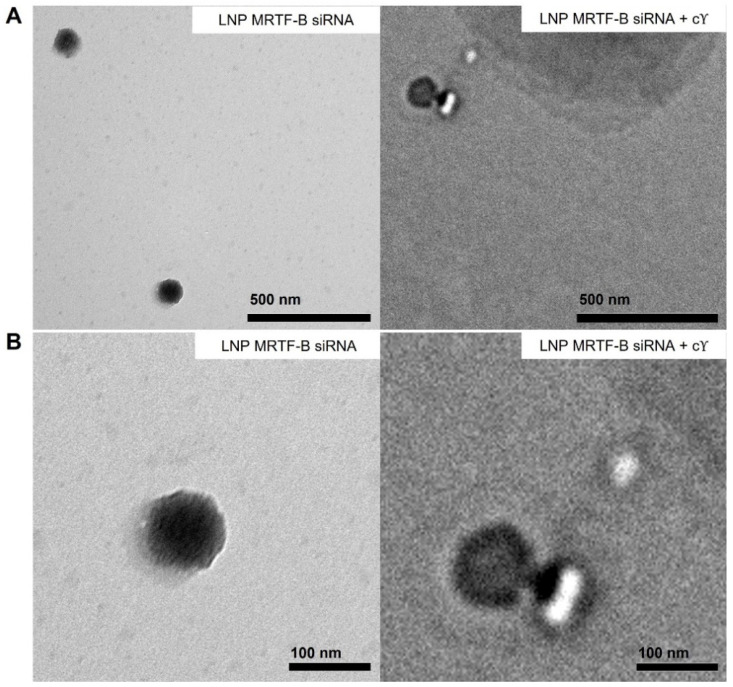
Negative staining transmission electron microscopy (TEM) images of LNP-MRTF-B siRNA and LNP-MRTF-B siRNA + cΥ. (**A**) Lower magnification. Scale bar, 500 nm. (**B**) Higher magnification. Scale bar, 100 nm.

We also studied the morphology of LNP-MRTF-B siRNA and LNP-MRTF-B siRNA + cΥ using negative staining TEM (Figure 3). The LNPs were spherical in morphology and the size of the two LNPs matched the results of the nanoparticle sizes shown in Figure 2A, confirming that the formulations were about 100 nm.

### 3.3. Gene Expression of MRTF-B, ACTA2, and COL1A2 in Human TM and FF Cells

**Figure 4:**
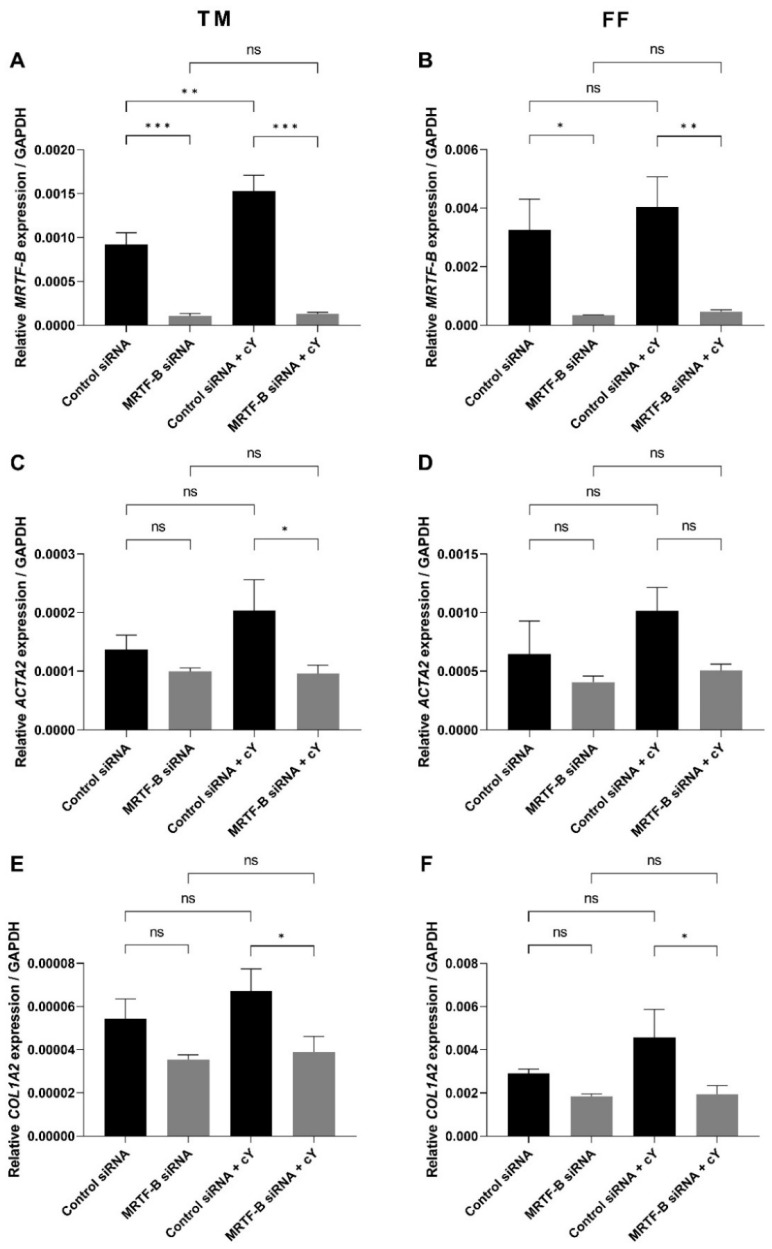
Gene expression of *MRTF-B*, *ACTA2*, and *COL1A2* in human TM and FF cells after transfection with four lipid nanoparticles (LNP-Control siRNA, LNP-MRTF-B siRNA, LNP-Control siRNA + cΥ, LNP-MRTF-B siRNA + cΥ). (**A**) *MRTF-B* expression in TM cells. (**B**) *MRTF-B* expression in FF cells. (**C**) *ACTA2* expression in TM cells. (**D**) *ACTA2* expression in FF cells. (**E**) *COL1A2* expression in TM cells. (**F**) *COL1A2* expression in FF cells. Results represent mean ± SEM. *n* = 3. *, *p* < 0.05; **, *p* < 0.01; ***, *p* < 0.001; ns, not significant.

The silencing of *MRTF-B* gene expression in human TM and FF cells was measured after treatment with the MRTF-B siRNA LNPs. The relative mRNA expression of *MRTF-B* gene in TM and FF cells treated with LNP-MRTF-B siRNA at an siRNA concentration of 50 nM decreased by 88.3% and 89.7%, respectively. The decrease was statistically significant for both TM (*p* < 0.001) and FF cells (*p* = 0.02). On the other hand, when treated with LNP-MRTF-B siRNA + cΥ, *MRTF-B* gene expression was significantly reduced by 91.5% (*p* < 0.001) and 88.6% (*p* = 0.009) in both TM and FF cells, respectively. Overall, the addition of cΥ further increased the *MRTF-B* gene silencing from 88.3% to 91.5% in the TM cells but not in the FF cells (Figure 4A,B).

The expression level of *ACTA2* gene in TM and FF cells treated with LNP-MRTF-B siRNA was also reduced by 27.5% (*p* = 0.40) and 36.8% (*p* = 0.37), respectively, compared to LNP-Control siRNA. Meanwhile, LNP-MRTF-B siRNA + cΥ reduced the gene expression of *ACTA2* by 52.8% (*p* = 0.03) and 50.2% (*p* = 0.08) in both TM and FF cells, respectively, compared to LNP-Control siRNA + cΥ. The addition of cΥ further decreased *ACTA2* gene expression in both TM and FF cells, and with statistical significance for TM cells (Figure 4C,D).

*COL1A2* is another downstream gene of the MRTF-B/SRF pathway. Compared to LNP-Control siRNA, the gene expression level of *COL1A2* in TM and FF cells treated with LNP-MRTF-B siRNA decreased by 35.0% (*p* = 0.13) and 37.0% (*p* = 0.30), respectively. LNP-MRTF-B siRNA + cΥ reduced *COL1A2* gene expression by 42.2% (*p* = 0.03) and 57.4% (*p* = 0.03) in TM and FF cells, respectively, compared to LNP-Control siRNA + cΥ. The addition of cΥ increased the downregulation of *COL1A2* gene in both TM and FF cells, and with statistical significance (Figure 4E,F).

### 3.4. Cell Viability of Human TM and FF Cells following Treatment with LNP-siRNAs

**Figure 5:**
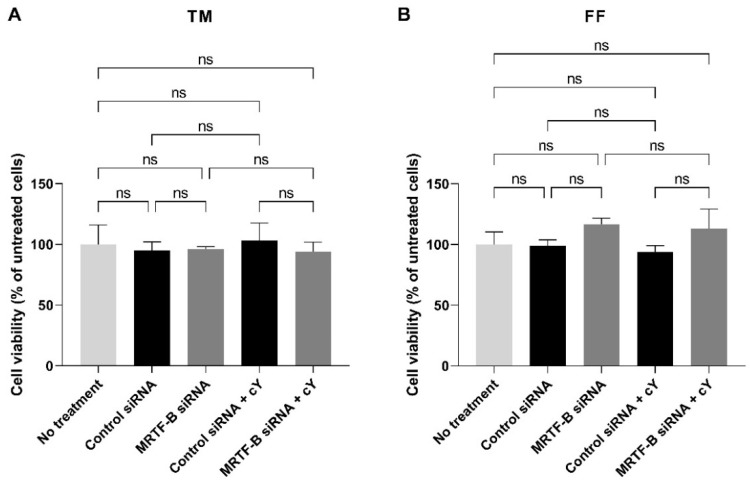
Cell viability of human (**A**) TM and (**B**) FF cells after 48 h post-transfection with four lipid nanoparticles (LNP-Control siRNA, LNP-MRTF-B siRNA, LNP-Control siRNA + cΥ, LNP-MRTF-B siRNA + cΥ) at 50 nM siRNA concentration. Data were normalised against untreated cells. Results represent mean ± SEM. *n* = 3. ns, not significant.

In the TM cells, at an siRNA concentration of 50 nM, the cell viability was similar after transfection with all four LNP-siRNAs. Compared to the untreated cells, the mean cell viabilities of TM cells treated with LNP-Control siRNA, LNP-MRTF-B siRNA, LNP-Control siRNA + cΥ, and LNP-MRTF-B siRNA + cΥ were 94.9 ± 7.1% (*p* > 0.99), 96.3 ± 1.9% (*p* > 0.99), 103.4 ± 14.2% (*p* > 0.99), and 94.1 ± 7.6% (*p* > 0.99), respectively. The addition of cΥ had no significant effect on the cell viability of TM cells (Figure 5A).

Similarly, the cell viability in the FF cells treated with four LNP-siRNAs at an siRNA concentration of 50 nM showed no statistical difference when compared to the untreated cells. The mean cell viabilities of FF cells treated with LNP-Control siRNA, LNP-MRTF-B siRNA, LNP-Control siRNA + cΥ, and LNP-MRTF-B siRNA + cΥ were 99.0 ± 4.8% (*p* > 0.99), 116.7 ± 5.0% (*p* = 0.72), 93.8 ± 5.2% (*p* = 0.99), and 113.2 ± 16.0% (*p* = 0.85), respectively. The addition of cΥ also had no significant effect on the cell viability of FF cells (Figure 5B).

### 3.5. Contractibility of Human TM and FF Cells Transfected with LNP-siRNAs

**Figure 6:**
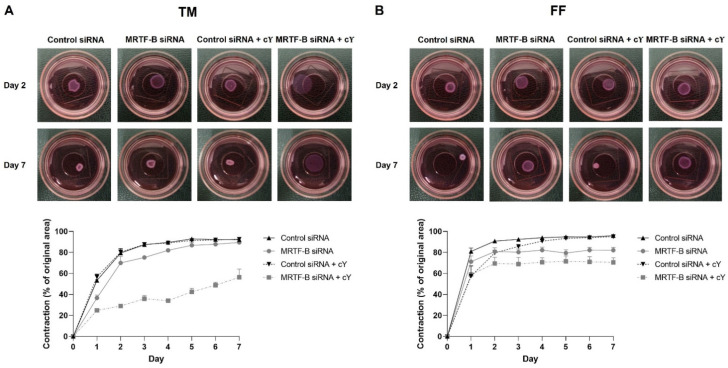
Three-dimensional collagen contraction assay of human (**A**) TM and (**B**) FF cells after transfection with four lipid nanoparticles (LNP-Control siRNA, LNP-MRTF-B siRNA, LNP-Control siRNA + cΥ, LNP-MRTF-B siRNA + cΥ) throughout the 7 days. Representative gel areas on day 2 and day 7 are shown.

**Figure 7:**
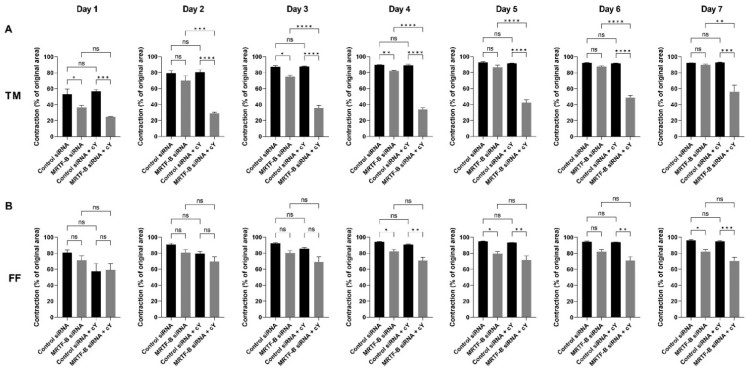
Collagen contraction assay of human (**A**) TM and (**B**) FF cells after transfection with four lipid nanoparticles (LNP-Control siRNA, LNP-MRTF-B siRNA, LNP-Control siRNA + cΥ, LNP-MRTF-B siRNA + cΥ) throughout the 7 days. The percentage contraction was calculated from the original area on day 0. Results represent mean ± SEM. *n* = 3. *, *p* < 0.05; **, *p* < 0.01; ***, *p* < 0.001; ****, *p* < 0.0001; ns, not significant.

Cell contractibility, another indicator of the efficiency of *MRTF-B* gene silencing, was measured using a three-dimensional cell-populated collagen contraction assay *in vitro*. All the cell-populated collagen gels containing human TM or FF cells, that were transfected by the four LNP-siRNAs, had contracted after 7 days from the start of the assay (Figure 6 and Figure 7).

The percentage contraction of TM-collagen gels transfected by LNP-Control siRNA, LNP-MRTF-B siRNA and LNP-Control siRNA + cΥ significantly increased on the first few days and stabilised in the last three days, while the percentage contraction of TM-collagen gel transfected by LNP-MRTF-B siRNA + cΥ had the lowest matrix contraction throughout the 7 days (Figure 6A). The TM-collagen gel transfected by LNP-MRTF-B siRNA with or without cΥ contracted less than the TM-collagen gel transfected by LNP-Control siRNA with or without cΥ throughout the 7 days (Figure 6A). The differences in matrix contraction between LNP-MRTF-B siRNA + cΥ and LNP-Control siRNA + cΥ were statistically significant on every day throughout the 7 days, whereas the statistical significance in matrix contraction between LNP-MRTF-B siRNA and LNP-Control siRNA only occurred on days 1, 3, and 4 (Figure 7A). The addition of cΥ in LNP-MRTF-B siRNA + cΥ made the TM-collagen gel contract less than LNP-MRTF-B siRNA without cΥ throughout the 7 days, with statistical significance on every day from days 2 to 7 (Figure 7A). On day 7, the LNP-MRTF-B siRNA + cΥ-transfected TM-collagen gel contracted 56.4 ± 7.9%, which was 39.2% less than the LNP-Control siRNA + cΥ and 37.1% less than the LNP-MRTF-B siRNA without cΥ. Although the LNP-MRTF-B siRNA without cΥ also contracted less than the LNP-Control siRNA, it was only 2.8% smaller (Figure 6A and Figure 7A).

For FF cells, the contraction area significantly increased on the first two days and stabilised for the following five days for all four LNP-siRNAs (Figure 6B). The differences between LNP-MRTF-B siRNA and LNP-Control siRNA were statistically significant on days 4, 5 and 7, whereas the differences between LNP-MRTF-B siRNA + cΥ and LNP-Control siRNA + cΥ were statistically significant on every day from days 4 to 7 (Figure 7B). There were no statistically significant differences between the presence of cΥ and the absence of cΥ in FF cells, which was different from the TM cells (Figure 7B). On day 7, the LNP-MRTF-B siRNA + cΥ-treated FF-collagen gel showed the lowest matrix contraction with a value of 70.5 ± 4.4%, which was 25.8% less than the LNP-Control siRNA + cΥ-treated FF-populated collagen gel but still more than that of the corresponding TM-populated collagen gel. Additionally, the FF-collagen gel transfected by LNP-MRTF-B siRNA without cΥ contracted 82.1 ± 2.7%, which was 14.4% less than the LNP-Control siRNA (Figure 6B and Figure 7B).

## 4. Discussion

Over the past decade, the development and clinical use of MIGS have revolutionised surgical options towards lowering IOP in glaucoma patients, by offering a better safety profile and promoting the AH outflow via an ab interno or an ab externo approach, with little or no scleral dissection and minimal or no conjunctival manipulation [33]. Despite being relatively safe and demonstrating few serious adverse events, the long-term success rate of MIGS is low and needs to be further improved. Studies have reported that only 50.0% of 60 eyes with iStent inject, 39.7% of 73 eyes with Hydrus, and 41.2% of 34 eyes with Kahook Dual Blade achieved an IOP reduction of 20% after 1-year follow up [34,35,36]. Similar to trabeculectomy, fibrosis is the main cause of surgical failure in MIGS. Scarring and fibrotic membranes are observed around failed CyPass, iStent inject, Hydrus implants as well as in failed Kahook Dual Blade surgery [6,7].

Currently, there are no anti-fibrotic treatments available for intraocular use in MIGS. The antimetabolites, MMC and 5-FU, are current gold standards in glaucoma filtration surgery, but lead to potentially sight-threatening complications, such as tissue damage, breakdown and severe infection, and thus cannot be used inside the eye [37]. Monoclonal antibodies, such as the anti-TGF-β2 antibody, are one of the largest classes of therapeutic molecules with the advantage of target specificity, but their anti-fibrotic efficacy in clinic remains to be further confirmed [38]. Another therapeutic approach, small molecule inhibitors, such as pirfenidone that downregulates a series of key profibrotic cytokines and growth factors, are still in the experimental research stage [39]. Among these innovations, nanotechnology and nanoparticles represent an area of great research interest in clinical translation due to their large surface area, good penetration, improved bioavailability, and ability for targeted drug delivery [40].Treatments based on siRNA-mediated gene silencing have shown promising outcomes in a variety of ocular diseases, including glaucoma [41]. To safely and efficiently deliver siRNAs and to release them at pertinent sites within targeted cells, nanotechnology is applied in the formulation of non-viral siRNA vectors [42]. We have previously achieved efficient *MRTF-B* gene silencing in human conjunctival fibroblasts (FF) by utilising novel PEGylated CL4H6-MRTF-B siRNA-loaded LNPs with no cytotoxicity [28]. In this study, we further tested these LNPs in human TM cells *in vitro* and compared their effects with those on FF cells, demonstrating proof-of-concept for a non-viral gene therapy in MIGS.

Appropriate biophysical properties allow the LNPs to entrap a large number of siRNAs and protect them from enzymatic degradation and rapid elimination, which are closely related to the transfection efficiency and cytotoxicity [40,43,44]. All four LNPs used in this study were spherical with a size under 100 nm and a nearly neutral or weakly cationic charge [45]. Size and charge play an important role in the cellular uptake and cytotoxicity of nanoparticles [46]. In this study, the size of nanoparticles can potentially enhance the retention and permeability of siRNAs [41], and their near neutral charge can be beneficial for the PEGylated formulation to achieve superior gene silencing, as the PEGylation of the interfaces might impact membrane flexibility and ensure steric stabilisation of the nanoparticles [24,29,47]. The PDI of the LNPs was also less than 0.3, which represented a homogenous particle size distribution. Moreover, all LNPs achieved high encapsulation efficiencies of over 90%.

Cell viability reflects the cytotoxicity and safety of therapeutic modalities. The LNPs in this study caused no significant cell death in both human TM and FF cells at an siRNA concentration of 50 nM. Our previous studies observed significant cytotoxicity in human FF cells at a higher siRNA concentration of 100 nM [28,48]. 50 nM is a viable concentration for siRNA delivery in human cells *in vitro*, however, whether it is an ideal dosage for *in vivo* research and the relationship between LNP-siRNA concentration, cytotoxicity, and gene silencing efficiency needs to be further investigated in animal models.

The therapeutic target of siRNA is the MRTF-B/SRF pathway that upregulates ocular fibrosis. The PEGylated CL4H6-LNPs successfully delivered the MRTF-B siRNA *in vitro* and achieved very high *MRTF-B* gene silencing of 88.3% and 91.5% without and with cΥ, respectively, in TM cells, and 89.7% and 88.6% in FF cells. This is the first study using non-viral siRNA gene therapy in human TM cells and it has shown significant gene silencing efficacy. We also measured the expression of downstream genes of the MRTF-B/SRF pathway. *ACTA2* is the encoding gene of α-smooth muscle actin (α-SMA), and *COL1A2* gene encodes one of the ECM proteins, type I collagen α2 chain [49,50]. Fibrosis is characterised by excessive accumulation of collagenous proteins in the ECM [51]. High levels of α-SMA due to either external mechanical force or the stimulation of TGF-β1 in myofibroblasts can initiate ECM stress [51,52]. In this study, both genes were downregulated after treatment with MRTF-B siRNA-loaded LNPs in TM and FF cells, especially in the presence of cΥ, which further confirmed the inhibition of the MRTF-B/SRF pathway and its downstream myofibroblast and cytoskeletal gene expression.

The lower *MRTF-B* gene expression was linked to the inhibition of matrix contraction in the MRTF-B/SRF downstream signaling, which was demonstrated in the contraction assay performed in both TM and FF cells. The collagen contraction assay is a three-dimensional functional assay that helps to visualise the dynamic interactions between ECM and cells [53], and all current gold standard treatments have previously shown efficacy using this assay. Resident cells actively and continuously remodel the ECM and can respond to biochemical cues of the ECM, thereby creating a state of dynamic equilibrium [53]. In this study, both TM and FF cells contracted significantly less after *MRTF-B* gene silencing, indicating a decrease in fibrosis development.

Another interesting finding is that the presence of cΥ led to increased inhibition of matrix contraction in TM cells, but not in FF cells [28]. We added cleavable cΥ peptide to test its effects on the biophysical properties, cytotoxicity and transfection efficiency of LNPs. In this study, the LNPs became smaller and more cationic with the addition of cΥ. Overall, there was no significant effect on encapsulation efficiency and cytotoxicity, but more importantly, the addition of cΥ increased *MRTF-B* gene silencing efficiency from 88.3% to 91.5% when compared to the corresponding LNP without cΥ. Nucleic acids, including plasmid DNA, mRNA, miRNA, and siRNA, share similar chemical components that define their hydrophilic and negatively charged nature [54]. The peptide cΥ has a positively charged RNA binding domain, which helps to attract multiple siRNAs. The electrostatic interactions force the LNPs to be tightly packed and prevent premature leakage of siRNAs from the LNPs [28,54], which might be the reason of the smaller LNP size in the presence of cΥ. Due to the cleavage of the peptide, the LNPs are also more easily detached and released from the receptor [55]. All these factors contributed to the increase in gene silencing efficiency and led to the highest decrease in contractibility of TM cells transfected with the LNP-MRTF-B siRNA + cΥ.

Furthermore, the properties of LNPs suggest its potential use as an intraocular drug-eluting delivery device, which is an innovative technology in glaucoma management. Currently, only one sustained-release glaucoma medication is approved by the FDA, the bimatoprost implant (Durysta, Allergan) [56], and two surgically implanted devices are currently in clinical trials, the ENV515 (Envisia Therapeutics) and iDose (Glaukos) [57]. The LNPs are thus expected to have broader future applications.

Further investigations on the administration route and kinetics of the LNPs will be our next steps for clinical translation in the future. Previous studies have reported the possibility of both subconjunctival and intravitreal injections of nanoparticles [29,58,59]. Several studies also focus on the distribution and clearance of nanoparticles in the eye. Shi et al. showed that RNA nanoparticles entered the conjunctiva, cornea, retina, and sclera after subconjunctival delivery. About 6–10% of the larger nanoparticles remained in the eye, and up to 70% of the retinal cells contained the nanoparticles at 24 h after administration [60]. Kim et al. also found that the nanoparticles migrated posteriorly to the retina 7 days after an intravitreal injection, as well as anteriorly into the aqueous humour from 1 h to 1 day after injection. Nanoparticles accumulated in the internal limiting membrane with no penetration into deeper retina, whereas the smaller nanoparticles moved through the ciliary body and reached the choroid, retina and suprachoroidal space [59].

Our study has a few limitations. Although the primary FF cell line used in this study is a good *in vitro* model to study conjunctival fibrosis after glaucoma surgery [28], it was cultured from only one patient. Besides the cell morphology, a panel of markers could be used to differentiate TM cells, such as chitinase-3 like-1 and myocilin [61]. Additionally, the cell viability method used in this study is a well-established method to measure cytotoxicity, but the evaluation of annexin V-positive cells could be carried out in the future for apoptosis analysis.

In summary, we demonstrate for the first time that the near neutral PEGylated lipid nanoparticles successfully delivered the MRTF-B siRNA into human TM cells *in vitro* and achieved significant gene silencing of *MRTF-B*, as well as a significant reduction in cell contractibility with no associated cytotoxicity. The addition of cΥ further increased the gene silencing efficiency and significantly reduced the contractibility of TM cells. LNPs can thus serve as a promising non-viral gene therapy to prevent fibrosis in MIGS.

## Figures and Tables

**Table 1:** Structures of the different lipids and sequences of the peptide and siRNAs.

Function	Name	Structure/Sequence
Lipid	7-(4-(dipropylamino)butyl)-7-hydroxytridecane-1,13-diyl dioleate (CL4H6)	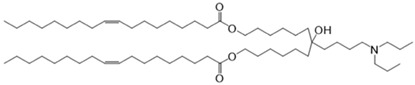
Lipid	1,2-dioleoyl-sn-glycero-3-phosphoethanolamine (DOPE)	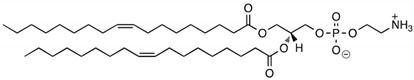
Lipid	1,2-dimirystoyl-rac-glycero, methoxyethylene glycol 2000 ether (PEG-DMG)	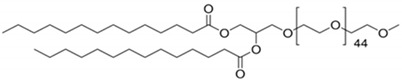
Peptide	Cleavable Υ (cΥ)	K_16_RVRR-GACYGLPHKFCG
siRNA	MRTF-B	Sense: GGAUGGAACUUUACCCUCA Antisense: UGAGGGUAAAGUUCCAUCC
siRNA	Irrelevant Control	Sense: UGGUUUACAUGUCGACUAA Antisense: UUAGUCGACAUGUAAACCA

## Data Availability

Not applicable.
